# Resilience Predicts Lower Anxiety and Depression and Greater Recovery after a Vicarious Trauma

**DOI:** 10.3390/ijerph182312608

**Published:** 2021-11-30

**Authors:** Christophe Leys, Ilios Kotsou, Rebecca Shankland, Mathilde Firmin, Sandrine Péneau, Pierre Fossion

**Affiliations:** 1 Faculty of Psychological Sciences, Université Libre de Bruxelles, 1050 Brussels, Belgium; ilios.kotsou@ulb.be (I.K.); firminmathilde1@gmail.com (M.F.); pierre.fossion@gmail.com (P.F.); 2 Education and Vulnerabilities, Laboratory DIPHE (Development, Individual, Personality, Handicap, Education), Department of Psychology of Development, University Lumière Lyon 2, 69000 Lyon, France; 3 INSERM U1153, INRAE U1125, CNAM, Nutritional Epidemiology Research Team (EREN), Epidemiology and Statistics Research Center, University of Paris (CRESS), Sorbonne Paris Nord University, 94000 Bobigny, France; s.peneau@uren.smbh.univ-paris13.fr

**Keywords:** resilience, vicarious trauma, brief resilience scale, French validation, anxiety, depression

## Abstract

This study validated the French version of the Brief Resilience Scale (BRS-F) and tested the protective role of resilience in the context of vicarious trauma (22 March 2016 terrorist attacks in Brussels) regarding anxiety and depression symptoms. Confirmatory factor analyses indicated a single-factor structure of the BRS-F. Investigation of convergent validity showed that the BRS-F was positively correlated with usual outcomes such as subjective happiness, acceptance, and sense of coherence, and negatively correlated with anxiety and depression symptoms. Lastly, the results of the study showed that resilience protected against the effect of vicarious trauma in two ways. First, at the time of exposure, the more resilient individuals reported lower levels of anxiety and depression symptoms. Second, after three months, the more resilient individuals recovered from these symptoms, whereas no significant effect was found for less resilient individuals. Theoretical and clinical implications of the findings are discussed.

## 1. Introduction

According to Luthar et al. [1], resilience is a dynamic process including positive adaptation in a context of significant adversity. Resilience is associated with many crucial stakes in terms of public health [2]. Indeed, resilience is inversely correlated with major psychiatric syndromes such as anxiety and depression [3], post-traumatic stress disorders [4], eating disorders [5], and addictions [6]. Hence, if bolstering resilience diminishes different forms of psychological distress, it is also essential to study in the context of vicarious trauma.

The diversity of the elements that potentially determine resilience in various contexts as well as the number of scales used to measure resilience contributes to the difficulty in understanding how resilience contributes to mental health. In their review, Ahern et al. [7] highlighted six main scales used to measure resilience: the Brief Resilient Coping Scale (BRCS, [8]), which describes resilience as a one-factor concept; the Resilience Scale for Adult-RSA [9], including six factors; the Resilience Scale-RS [10], including two factors; the Adolescent Resilience Scale-ARS [11], including three factors; the Baruth Protective Factors Inventory-BPFI [12], including four factors; and, finally, the Connor-Davidson Resilience Scale-CD-Risk [13], which comprises five factors. Despite the diversity of dimensional structures and scales, they generally share common dimensions related to the individual, their social environment, their family environment, and acceptance (as is the case for RS and CD-Risk). Acceptance is defined as “the willingness to experience” (i.e., not alter the form, frequency, or sensitivity of) unwanted private events, in order to pursue one’s values and goals (e.g., being willing to feel fear in pursuit of a long-held goal [14]).

With regard to the definition of resilience, Smith et al. [15] published an article underlining the importance of evaluating the core definition of resilience, which is the ability to bounce back from stress or trauma. Considering this, they validated in the same article the Brief Resilience Scale (BRS), which specifically aims to evaluate resilience as the ability to bounce back from stress or trauma. Their study showed that the BRS presented a one-factor solution, demonstrating a good internal consistency and test–retest reliability.

In terms of convergent validity, the BRS was positively correlated with resilience resources, measured by the Connor–Davidson Resilience Scale [13], the Ego Resiliency Scale [16], and the Brief COPE [17], and was positively correlated with good physical and psychological health outcomes, evaluated by the Hospital Anxiety and Depression Scale [18], the Mental Health Inventory [19], the Mood Adjective Checklist [20], the Physical Symptoms Index [21], and the Perceived Stress Scale [22]. Other authors, such as Jordan [23], described different factors, including resilience, which buffers the symptoms of vicarious trauma among military and civilian therapists working with combat veterans. In the same way, Vrklevski and Franklin [24] showed that subjects sustaining a vicarious trauma (i.e., law solicitors frequently witnessing their clients’ distress) were likely to develop anxiety and depression symptoms. Lastly, adapted coping strategies, closely related to resilience, such as exercising, meditating, or seeking peer support, were likely to help subjects to overcome these symptoms.

The aim of the present study was twofold: first, to validate a French version of the BRS (BRS-F); second, to test the protective role of resilience in the context of vicarious trauma (22 March 2016 terrorist attacks in Brussels), regarding anxiety and depression symptoms. To achieve the first goal and validate the BRS-F, we aimed at confirming the correlations between resilience and usual outcomes such as subjective happiness, acceptance, sense of coherence, and anxiety and depression symptoms.

Subjective happiness refers to a positive evaluation of one’s life and the presence of positive emotions, which is in line with the hedonic conception of well-being [25]. Gomez et al. [26] showed that subjective happiness was positively correlated with the CD-RISK resilience scale (r = 0.46).

Acceptance is a measure of psychological flexibility/inflexibility that are central components in Acceptance and Commitment Therapy [27]. Several studies underlined the link between acceptance and resilience. For example, Calvo et al. [28] highlighted a significant positive correlation between acceptance and the Resilience Scale for Adults (r = 0.72). In the same way, Meyer et al. [29] demonstrated a positive correlation between acceptance and the English version of the BRS (r = 0.61).

The sense of coherence was conceptualized by Antonovsky [30] as a general orientation to view the world and the individual environment as comprehensible, manageable, and meaningful, claiming that the way people view their life has a positive influence on their health [4,31]. Fossion et al. [32] demonstrated a positive correlation between sense of coherence and the French version [33] of the Resilience Scale for Adults (r = 0.68).

Depression and anxiety have been frequently demonstrated to be negatively correlated with resilience. Specifically, Fossion et al. [3] showed this negative correlation between anxiety and depression symptoms and the Resilience Scale for Adults (r = −0.54).

We therefore hypothesized that (1) the BRS-F would correlate in the expected way with the outcomes previously described; (2) after a vicarious trauma (i.e., terrorist attacks), more resilient participants would display less anxiety and depression symptoms; (3) after a vicarious trauma, more resilient participants would recover faster from anxiety and depression symptoms than less resilient participants.

## 2. Materials and Methods

### 2.1. Participants and Procedure

The sample was composed of 464 participants (Mage = 47.24, SD = 12.58, 78% women) who responded at two time points for this study. This sample came from a larger database of 2077 participants that was collected the day following the terrorist attacks of 22 March 2016 in Brussels (Time 0). Among the 2077 participants, 1109 accepted participation in the follow-up study that we carried out and were contacted by email for the second measurement point aimed at testing resilience. In total, 464 of them (42%) completed the second survey (Time 1). Participants were asked to click on a specific button after having read the informed consent form in order to respond to the questionnaire. Among the 464 participants, 86% were Belgian, 9% French, and 5% came from other countries; 257 participants (55%) lived in a couple, 136 (29%) were single, and 71 (16%) were divorced; 324 participants (70%) were employees, 25 (5%) self-employed, 20 (4%) unemployed, 43 (9%) students, and 52 (11%) had another status; 233 participants (50%) were living in Brussels at that time, and 259 (56%) were present in Brussels during the attacks. Furthermore, 70 participants (15%) were using medication such as antidepressants, tranquilizers, or sleeping pills. An online questionnaire assessed several mental health outcomes and measures usually associated with resilience at Time 0. At Time 1, three months later, the level of resilience was also measured through a translated version of the Brief Resilience Scale (Supplementary Materials).

### 2.2. Measures

Brief Resilience Scale (BRS-F). The BRS-F [15] was translated into French by two co-authors (I.K., S.P.) and back-translated by two native English-speaking colleagues, among which was one co-author (R.S.), and corrected to reach an agreement between all the translators. This scale comprises 6 items rated using a Likert scale ranging from 1 (strongly disagree) to 5 (strongly agree; α = 0.86). The even items are then reverse coded. The higher the score, the greater the resilience.

Subjective Happiness Scale (SHS-F). SHS-F is a scale assessing subjective happiness developed by Lyubomirsky and Lepper [25] and validated in French [34]. It is a one-factor solution of a 4-item Likert scale (the third is reversed), ranging from 1 to 7 (α = 0.83). Scores range from 4 to 28, with higher scores indicating higher levels of subjective happiness.

The Acceptance and Action Questionnaire (AAQ-II). AAQ-II was developed by Bond et al. [35], and validated in French by Monestès et al. [36]. It is a 7-item Likert scale ranging from 1 to 7 with a one-factor solution (α = 0.92).

Sense of Coherence (SOC-13). The SOC-13 is a 13-item, 7-point Likert scale (α = 0.82) self-report questionnaire [37]. The French version of the SOC scale has satisfying reliability as well as convergent and discriminant validity [38].

Hopkins Symptom Checklist (HSC). The HSC [39] is a 25-item tool assessing the symptoms of depression and anxiety. This self-report questionnaire uses a 4-point Likert scale. This scale contains 15 depression items and 10 anxiety items. The HSC is highly reliable (α = 0.93) and is a valid diagnostic screening tool, even across cultures. A second-order factor is also used as valid measure of depressive and anxiety disorder symptoms. The French version of the HSC has been validated by previous studies [40].

## 3. Results

One-way ANOVAs were performed to check for the effects of social and demographic variables on the BRS-F. None of them showed significant effects. Therefore, these variables were not included in further analyses.

### 3.1. Confirmatory Factor Analysis

We first conducted a confirmatory factor analysis on two subsamples by randomly splitting our database in two subsamples using R (v.3.6.1). Each observation had a 0.5 probability to be included in a subsample. The first subsample included 215 participants, and the second, 249. A first confirmatory analysis (N = 215) determined the correlations that had to be estimated between item perturbations (When conducting a CFA, one can read the modification indices. It is a measure of the amount of the chi-square one would gain by estimating a parameter that is currently not estimated (i.e., set at 0). Each item is associated with a random error named “perturbation”. Such errors can be correlated between similar items since the error applied to a given item can be influenced by similar processes for similar items. In such a situation, failing to estimate such correlations and, by default, considering that they are independent (correlation set at 0) is not consistent with the data. This implies that one thus needs to estimate the correlation between those perturbations to fit the model with the data, at the cost of a degree of freedom spent to estimate the parameter). Preliminary results indicated that correlations of perturbations between items 3 and 5 and between items 1 and 5 needed to be estimated by the model. The original CFA yielded a good model fit: *χ*^2^(9) = 25.90, *p* = 0.002; CFI = 0.96; TLI = 0.94; RMSEA = 0.09 [0.05–0.014], *p* = 0.04. Following Kline [41], a good model fit implies a non-significant chi-square (ideally, or a ratio *χ*^2^/ddl < 3) and a CFI and TLI over 0.95, and an RMSEA is acceptable under 0.10, good under 0.08, and very good under 0.05. Estimating the two correlations between item perturbations improved the model: *χ*^2^(7) = 9.67, *p* = 0.21; CFI = 0.99; TLI = 0.99; RMSEA = 0.04 [0.00–0.010], *p* = 0.52. We applied the same model on the second subsample, which yielded a very good model fit: *χ*^2^(7) = 12.05, *p* = 0.10; CFI = 0.99; TLI = 0.98; RMSEA = 0.05 [0.00–0.010], *p* = 0.39. Table 1 and Table 2 report descriptive statistics of all the variables at Time 0 and Time 1, and Table 3 reports correlations between the BRS-F and the outcomes at Time 2. As suggested in hypothesis 1, all correlations were consistent with previous findings.

### 3.2. Protective Role of Resilience

The correlations presented in Table 3 indicate that more resilient individuals showed less anxiety and depression symptoms at Time 1 (three months after the event). To test hypothesis 2, we used a regression analysis predicting depression and anxiety symptoms at time 1 by resilience scores, while controlling for time 0. This analysis (R^2^ = 0.597) yielded a significant effect of HSC-T0 on HSC-T1 (*β* = 0.56, SE = 0.034, *p* < 0.001), but once HSC-T0 was controlled for, BRS significantly predicted HSC-T1 (*β* = −0.20, SE = 0.022, *p* < 0.001).

Hypothesis 3 stated that individuals higher in resilience would recover faster from depression and anxiety symptoms three months after a vicarious trauma. Consistent with this hypothesis, the results showed that resilience predicted the difference in HSC between T1 and T0 (*β* = −0.0447; SE = 0.0226; *p* = 0.048; R^2^ = 0.00845), indicating that the more resilient the individuals were, the faster they recovered, with a small effect size.

## 4. Discussion

The aim of this study was twofold: (1) to assess the psychometric properties of the French version of the Brief Resilience Scale (BRS-F), and (2) to analyze the protective role of resilience concerning mental health in the context of vicarious trauma. The results underlined that the psychometric properties of the BRS-F are satisfactory and measure the core of resilience through a brief 6-items questionnaire. The confirmatory factor analysis yielded satisfying fit index showing a one-factor structure. Moreover, the convergent validity was demonstrated through the correlations between BRS-F and many usually associated outcomes: sense of coherence, subjective happiness, acceptance, and psychological distress (anxiety and depression symptoms). These results thus encourage the use of this scale in future studies on French-speaking populations.

The results of our study also underlined the fact that resilience protects against the effect of vicarious trauma in two ways. First, at the time of exposure, the more resilient individuals reported lower levels of anxiety and depression symptoms. Second, after three months, resilient individuals recovered from these symptoms, whereas less resilient individuals did not show significant signs of recovery. Past research has shown that trait-resilience represents an important protection factor that helps individuals face adverse situations. It is useful to identify that this general ability to overcome difficult situations concerns both individuals who are implicated in a traumatic event and individuals who are only indirectly concerned. Indeed, research on vicarious trauma—also termed secondary trauma—has highlighted the fact that helpers may develop symptoms of trauma because of empathic engagement with clients who report their own trauma [42]. This “cost of care” highlighted by Figley [43] many years ago may be reduced through health promotion interventions that develop resilience. Indeed, in a study by Bober and Regehr [44], it was shown that usually recommended strategies (e.g., self-care, leisure, and supervision) for professionals working with clients having suffered from various traumas were ineffective. As past research has indicated that individual and situational factors (e.g., current life events, individual ability to tolerate strong affect, coping strategies, and personal history of trauma [45,46]) may exacerbate or reduce the risk of vicarious trauma, interventions have aimed at developing professional training to reduce vicarious trauma and increase resilience. It would be particularly useful to develop resilience during helpers’ initial training and measure it in order to identify helpers who may need more support to develop resilience and other mental health protection factors such as self-compassion, optimism, or hope. Resilience may be developed, for example, through mindfulness-based practices [47,48], positive psychology interventions [49], or compassion-focused therapy. Furthermore, by increasing professional skills to help the client overcome such a situation, it may help to reduce empathic distress fatigue by increasing the orientation of the professional towards helping relieve other’s suffering. This attitude has been termed “compassion” by Klimecki and Singer [50] and has been shown to be effective in reducing professional burnout rather than increasing it, contrary to empathic distress fatigue.

Although the results of our study are promising regarding the role of resilience in relation to vicarious trauma, some limitations of this study need to be underlined. First, the study only relied on self-report measures of mental health and resilience, which implies problems in interpreting the results as levels of awareness of one’s own psychological health, strengths, and difficulties vary between individuals. Further research is needed using complementary measures with close relatives’ reports and medical diagnosis of mental health symptoms. Another limitation is related to the data collection: in this study, we only had a measure of resilience after the stressful event. Previous authors have suggested that resilience might have two sides and can be considered as a trait but also as a skill [51]. In this regard, it has been underlined that resilience can be influenced by trauma [2,3] or enhanced by preparation [52] or training [53]. In the case of our study, it was not possible to assess this aspect. Furthermore, resilience was only measured at Time 1, while the participants might have experienced other traumatic events in the meantime. However, our analyses aimed at testing the relation between the level of resilience and the levels of depression and anxiety symptoms. This relation is well illustrated, even considering the limitation concerning the potential progress in resilience scores following the event.

## 5. Conclusions

This study highlighted the validity of the French version of the BRS as well as the protective role of resilience in the context of vicarious trauma. It underlined that resilience may act as a protection factor regarding anxiety and depression symptom recovery after having faced a vicarious trauma situation. It has meaningful implications for French-speaking researchers in the field of resilience. By validating BRS-F, we provide a new tool to assess the core of resilience, which is the ability to bounce back after a significant episode of stress. Moreover, the results of this study encourage further research on resilience related to vicarious trauma, especially among professionals who work with clients having faced traumatic events in order to reduce consecutive psychological distress [54]. The idea to prepare specific populations to face trauma is not new. Duagani Masika et al. [52] showed that prepared soldiers in the Republic of Rwanda could cope much better with trauma than unprepared populations facing comparable traumas. This preparation could also apply to helpers who may experience vicarious trauma. This may participate to the research agenda on resilience for healthcare and social workers.

## Figures and Tables

**Table 1:** Descriptive statistics at Time 0.

	AAQ-II	SHS	HSC_anx	HSC_dep	HSC_tot	SOC
N	464	464	464	464	464	464
Mean	3.20	4.91	1.86	1.86	1.86	4.52
Standard deviation	1.40	1.21	0.606	0.625	0.577	0.884
Minimum	1.00	1.00	1.00	1.00	1.00	1.00
Maximum	7.00	7.00	3.80	3.80	3.56	6.60

Note: AAQ-II = Acceptance and Action Questionnaire II; SHS = Subjective Happiness Scale; HSC_anx = Hopkins Symptom Checklist Scale anxiety; HSC_dep = Hopkins Symptom Checklist Scale depression; HSC_tot = Hopkins Symptom Checklist Scale total; SOC = Sense of Coherence Scale; BRS = Brief Resilience Scale.

**Table 2:** Descriptive statistics at Time 1.

	AAQ-II	SHS	HSC_anx	HSC_dep	HSC_tot	SOC	BRS
N	464	464	464	464	464	464	464
Mean	3.11	4.98	1.80	1.78	1.78	4.57	3.39
Standard deviation	1.27	1.21	0.58	0.62	0.57	0.94	0.86
Minimum	1.00	1.00	1.00	1.00	1.00	1.85	1.00
Maximum	7.00	7.00	3.90	3.80	3.68	6.69	5.00

Note: AAQ-II = Acceptance and Action Questionnaire II; SHS = Subjective Happiness Scale; HSC_anx = Hopkins Symptom Checklist Scale anxiety; HSC_dep = Hopkins Symptom Checklist Scale depression; HSC_tot = Hopkins Symptom Checklist Scale total; SOC = Sense Of Coherence Scale; BRS = Brief Resilience Scale.

**Table 3:** Correlations (Pearson’s r) between BRS and outcomes at Time 1.

	AAQ-II	SHS	HSC_an	HSC_dep	HSC_tot	SOC
SHS	−0.550	—				
HSC_anx	0.634	−0.405	—			
HSC_dep	0.693	−0.538	0.4	—		
HSC_tot	0.713	−0.518	0.903	0.963	—	
SOC	−0.726	0.589	−0.588	−0.714	−0.708	—
BRS	−0.661	0.518	−0.525	−0.585	−0.598	0.629

Note: AAQ-II = Acceptance and Action Questionnaire II; SHS = Subjective Happiness Scale; HSC_anx = Hopkins Symptom Checklist Scale anxiety; HSC_dep = Hopkins Symptom Checklist Scale depression; HSC_tot = Hopkins Symptom Checklist Scale total; SOC = Sense of Coherence Scale; BRS = Brief Resilience Scale. All *p*-values were < 0.001.

## Data Availability

Anonymized data are available online at the following address: https://osf.io/h5rcm/ (accessed on 20 November 2021).

## Supplementary Materials

The following are available online at https://www.mdpi.com/article/10.3390/ijerph182312608/s1, Brief Resilience Scale – French.
