# Supplementary material for: Resilience Predicts Lower Anxiety and Depression and Greater Recovery after a Vicarious Trauma
Source: Int J Environ Res Public Health. 2021 Nov 30;18(23):12608. doi: 10.3390/ijerph182312608 (PMC8656954; doi:10.3390/ijerph182312608)
Supplement: Supplementary file 1 [file ijerph-18-12608-s001.zip › ijerph-1396632-supplementary.pdf]

Supplementary Material

Brief Resilience Scale – French

Merci de bien vouloir indiquer ce qui vous correspond le mieux à chaque question sur une échelle de 1 (pas du tout d'accord) à 5 (tout à fait d'accord)

1. J'ai tendance à rebondir rapidement après les moments difficiles.
2. J'ai du mal à me sortir d'évènements stressants.
3. Je récupère rapidement après un évènement stressant.
4. J'ai du mal à revenir à mon état normal quand quelque chose de négatif arrive.
5. Je surmonte habituellement les moments difficiles avec peu de difficultés.
6. J'ai tendance à mettre longtemps pour me remettre des échecs dans ma vie.
